# Nutrients, Clock Genes, and Chrononutrition

**DOI:** 10.1007/s13668-014-0082-6

**Published:** 2014-04-27

**Authors:** Hideaki Oike, Katsutaka Oishi, Masuko Kobori

**Affiliations:** 1 Food Function Division, National Food Research Institute (NFRI), National Agriculture and Food Research Organization (NARO), 2-1-12 Kannondai, Tsukuba, Ibaraki 305-8642 Japan; 2 Biological Clock Research Group, Biomedical Research Institute, National Institute of Advanced Industrial Science and Technology (AIST), Central 6, 1-1-1 Higashi, Tsukuba, Ibaraki 305-8566 Japan; 3 Department of Medical Genome Sciences, Graduate School of Frontier Sciences, University of Tokyo, Kashiwa, Japan; 4 Department of Applied Biological Science, Graduate School of Science and Technology, Tokyo University of Science, Noda, Japan

**Keywords:** Circadian rhythm, Circadian clocks, Clock genes, Nutrition, Chrononutrition, High-fat diet, Metabolic disorders, Meal timing, Breakfast

## Abstract

Circadian clocks that comprise clock genes exist throughout the body and control daily physiological events. The central clock that dominates activity rhythms is entrained by light/dark cycles, whereas peripheral clocks regulating local metabolic rhythms are determined by feeding/fasting cycles. Nutrients reset peripheral circadian clocks and the local clock genes control downstream metabolic processes. Metabolic states also affect the clockworks in feedback manners. Because the circadian system organizes whole energy homeostasis, including food intake, fat accumulation, and caloric expenditure, the disruption of circadian clocks leads to metabolic disorders. Recent findings show that time-restricted feeding during the active phase amplifies circadian clocks and improves metabolic disorders induced by a high-fat diet without caloric reduction, whereas unusual/irregular food intake induces various metabolic dysfunctions. Such evidence from nutrition studies that consider circadian system (chrononutrition) has rapidly accumulated. We review molecular relationships between circadian clocks and nutrition as well as recent chrononutrition findings.

## Introduction

Life on earth proceeds with daily cyclic changes in circumstances. Plants conduct photosynthesis during the daytime, and nocturnal animals forage for food at night. Many living organisms have developed intrinsic 24-h cycles called circadian clocks that enable the expression of activities at appropriate times. The molecular mechanisms of clocks have been investigated in detail since the first clock mutant was isolated from fruit flies [1]. Several clock genes are homologous from flies to mammals and thus circadian clock systems in all vertebrates have the same origin, whereas plants, fungi, and protists have developed other circadian systems [2]. Regardless of the molecular bases, transcriptional-translational feedback loops play critical roles in the generation or maintenance of circadian rhythms. The main feedback loop in mammals comprises several core clock genes, including Bmal1, Clock, Per1/2, and Cry1/2 [3]. In addition to these, other clock genes or clock-controlled genes, such as Rev-erbα/β, Rorα/β, Dbp, Dec1/2, CK1ε/δ, and NPAS2 cooperate to sustain mammalian circadian clocks. Genome-wide transcriptome and ChIP-seq analyses have shown that clock genes control the transcription of thousands of genes with chromatin remodeling [4–6]. Notably, posttranscriptional regulation plays a substantial role in controlling circadian mRNA expression [4]. The circadian control of transcribed genes leads to rhythmic physiological events.

Light/dark cycles entrain the central clock in the suprachiasmatic nucleus (SCN) that is located in the hypothalamus where it mainly dominates activity-related rhythms, such as sleep/wake cycles, the autonomic nervous system, core body temperature, and melatonin secretion. In contrast, feeding/fasting cycles entrain peripheral clocks that are located in most tissues including even part of the brain [7•]. Peripheral clocks dominate local physiological processes, including glucose and lipid homeostasis, hormonal secretion, xenobiotics, the immune response, and the digestion system [8]. As the central clock organizes local clocks through neuronal and humoral signals, desynchronization among clocks is believed to result in the development of unpreferable conditions, such as metabolic disorders, cancer, and psychiatric disorders [9].

Circadian clocks enable the anticipation of daily events, conferring a considerable advantage for saving time and the efficient use of energy. The central clock activates the sympathetic nervous system and increases body temperature and blood pressure ahead of the active phase, facilitating the start of activities. Digestion/absorption systems also prepare before breakfast based on the time of local clocks [7•, 10]. Because colonic motility also is regulated by the local clocks, gastrointestinal symptoms are prevalent among shift workers and time-zone travelers [11]. In addition to local physiological events in tissues, some activity rhythms also are affected by feeding. Scheduled feeding elicits food anticipatory activity that is independent of light/dark cues and is perceived as food-seeking behavior approximately 2 hours before feeding [7•, 12]. This activity rhythm persists in rodents with SCN lesions, indicating that the central clock is not essential for food anticipatory activity. Because food available timing can be occasionally restricted in the wild, circadian anticipatory control of behavior and energy metabolism probably increases food usage and energy efficiency. Indeed, many studies have shown that circadian clocks intimately control energy metabolism [13]. Many genes associated with glucose and lipid homeostasis, especially those encoding rate limiting enzymes in various metabolic processes, are under circadian control. Thus, mutations or deletions of clock genes lead to metabolic disorders [14]. Mice with mutant Clock have attenuated feeding rhythm, hyperphagic, and obesity as well as altered gluconeogenesis, insulin insensitivities, and lipid homeostasis [15, 16]. Glucose and lipid homeostasis are similarly impaired in Bmal1 knockout mice [17, 18], and altered lipid metabolism, attenuated nocturnal food intake with total overeating, and developing significant obesity on high-fat diet are reported in Per2 knockout mice [19, 20]. A few studies have suggested an association between genetic variance in clock genes and metabolic risk in humans [14, 21•]. In addition, an epigenetic state of clock genes might be associated with obesity [22]. These genetic associations indicate mutual interaction among circadian clocks, metabolism, and nutrition.

**Fig. 1:**
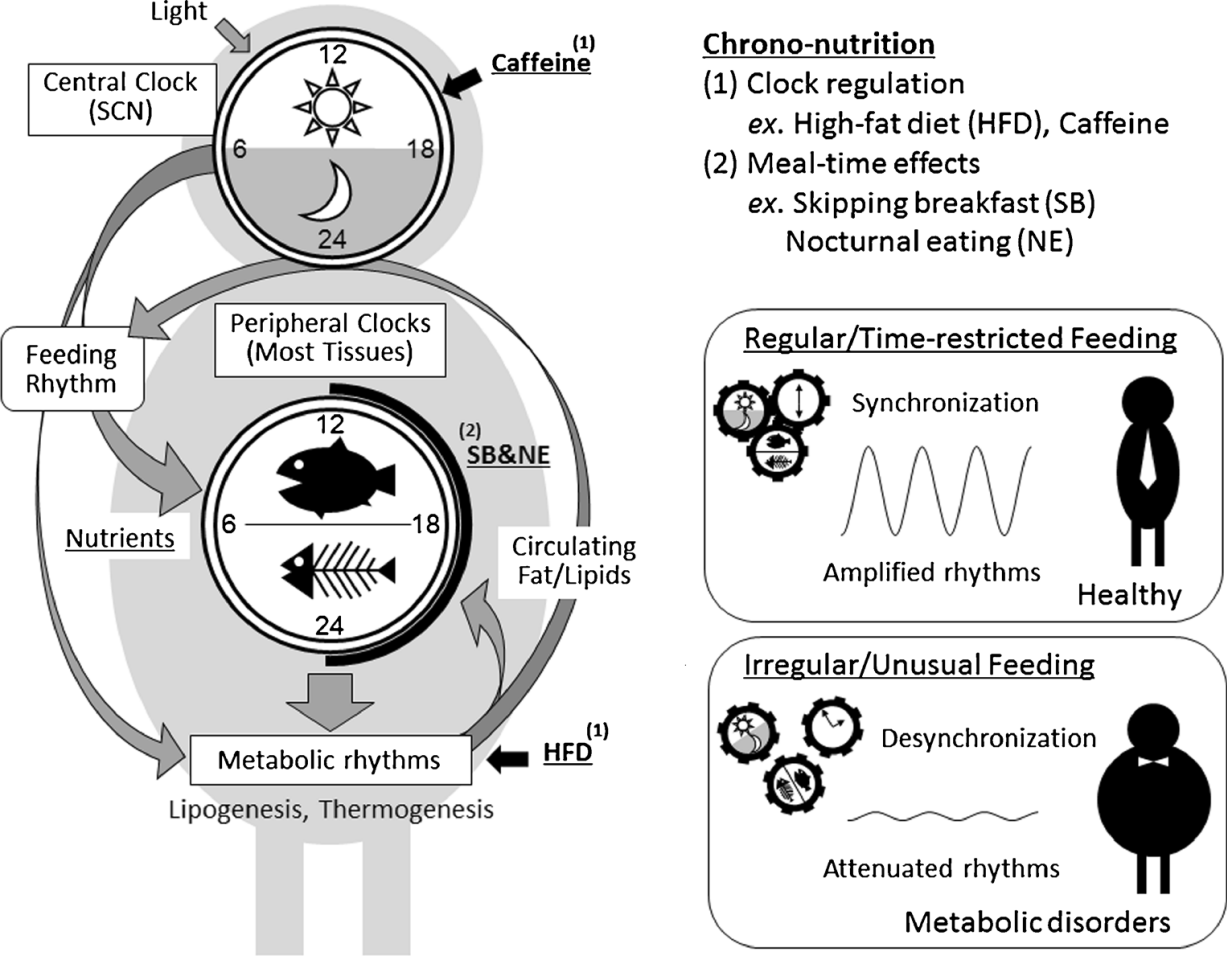
Schematic representation of the circadian clock system and chrononutrition. Light/dark cycles entrain the central clock in the suprachiasmatic nucleus (SCN) dominating activity rhythms, whereas feeding cues determine the phase of peripheral clocks that dominate local metabolic rhythms. Both nutrients and meal timing can affect the clock system, thus “chrononutrition” has two aspects: 1) nutrients/food components regulate the clock system, e.g., caffeine prolongs the period of circadian clocks and the locomotor activity rhythm, and high-fat diets alter the rhythms of lipogenesis, circulating lipids, locomotor activity, and feeding behavior; 2) meal-timing affects output of the clock system, e.g., skipping breakfast and nocturnal eating increases risk of obesity, whereas time-restricted feedings prevent metabolic disorders induced by high-fat diets. Regular/time-restricted feedings synchronize and amplify the rhythms of clock system, whereas irregular/unusual feedings cause desynchronization and attenuate the rhythms, probably leading to metabolic disorders

Recently, a novel field between nutrition and circadian clock system is referred as “chrononutrition” [7•, 10] (Fig. 1). In this article, we review recent findings regarding chrononutrition, food components that regulate circadian clocks, and meal times that affect metabolic homeostasis.

## Nutrients Reset Peripheral Circadian Clocks

Feeding time is a dominant factor in determining the phase of peripheral circadian clocks. Time-restricted feeding during the daytime for a week completely alters the phase of the circadian expression of clock and clock-controlled genes in the peripheral tissues of nocturnal rodents, whereas the central clock that is dominated by light/dark cycles is not affected [23–25]. The liver can adapt to novel feeding times within approximately 3 days, whereas the kidney, heart, pancreas, and lung take longer [23, 24]. Because liver clocks are rapidly entrained by feeding signals, many investigators have used the liver to clarify the features and mechanisms of food entrainment. It is revealed that the balance between food volume and starvation intervals is an important factor to determine the phase of the liver clock. Breakfast is usually the most effective meal to determine the phase of the liver clock in studies of mice that mimic human eating patterns, because breakfast is consumed after the longest starvation during the day [26]. Thus, late dinners or midnight snacks alter the starvation period and remarkably alter the phase of peripheral clocks [27]. The nutrients responsible for the rapid phase-shift in the liver clock also were investigated and revealed that a combination of carbohydrate and protein is essential to reset liver clocks, whereas either protein, sugar, or oil is insufficient [28]. Coincidently, liver clocks can be reset by an intraperitoneal injection of glucose combined with amino acids but not by either alone [29]. As blood glucose uptake after nutrient intake correlates with the amount of phase-shift in the liver when protein is included in the food, rapidly digestible starches with a high glycemic index powerfully entrain liver clocks [30]. The molecular mechanisms underlying the rapid phase-shift in the liver clock caused by nutrients has gradually been elucidated. Thirty minutes of feeding after a long interval rapidly induces the transient expression of Per2 and Dec1 genes and a shift in the phase of the clock in rat livers [31]. Feeding after a fast increases Per2 and decreases Per1 [32], increases Per2 and decreases Rev-erbα within 2 hours [33], and increases Per1, Per2 and Dec1 while decreasing Rev-erbα within 1 hour in the mouse liver [29]. These findings indicate that nutrients containing glucose and amino acids induce rapid changes in the expression of clock genes, especially Per2 and Rev-erbα, resulting in a phase-shift. Because Per2 induction and Rev-erbα reduction after food intake is not induced in streptozotocin (STZ)-treated insulin-deficient mice, insulin is a candidate anticipant that can reset peripheral clocks by feeding cues [33]. Indeed, an injection of insulin elicits a rapid change in Per2 and Rev-erbα expression and shifts the phase of liver clocks. Insulin added to cultured rat hepatocytes also induces rapid expression of Per1, Per2, and Dec1 genes [34]. The induction of clock genes is partially blocked by PD98059 (MAPK inhibitor) or LY294002 (PI3K inhibitor), suggesting that MAPK and PI3K are involved in resetting clocks downstream of insulin signaling. In addition to the liver, clock genes are rapidly induced by insulin injection in insulin-sensitive tissues, such as muscle and adipose, but not in insulin-insensitive tissues, such as the lung and brain, suggesting that insulin is involved in rapid resetting of the clock by nutrients in some peripheral tissues [34]. On the other hand, peripheral clocks, including the liver, can be still phase-shifted by daytime restricted feeding for several days even in STZ-treated insulin-deficient rats and mice [35, 36]. Factors other than insulin must be involved in the entrainment of peripheral clocks by nutrient cues and body temperature and serum response factor have the potential [10].

## Food Factors Modulate Circadian Clocks

Feeding mice with a high-fat diet *ad libitum* under constant darkness prolongs the period of circadian locomotor rhythm within a few weeks [37]. Under normal light/dark conditions, a high-fat diet attenuates the amplitude of day/night feeding and clock gene expression rhythms in adipose tissues and the liver. Other studies have shown that a high-fat diet induces a phase-advance of liver clocks and altered feeding rhythms [38••, 39]. These findings indicate that a high-fat diet affects the central clock and/or eating behavior. Unlikely to the high-fat diet, intake of a ketogenic diet, which comprises high-fat with low-carbohydrate contents, shortens circadian locomotor activity rhythm under constant darkness and the expression rhythm of clock and clock-controlled genes are phase-advanced in peripheral tissues under a normal light/dark condition [40]. Interestingly, ketogenic diets were designed to mimic the physiological response to starvation, and hypocaloric conditions induce a phase-advance in circadian locomotor activity rhythms [41]. Free fatty acids that are increased by a ketogenic diet in plasma might be key factors that affect clocks because the intake of bezafibrate, a PPARα agonist that is activated by free fatty acids, induces a phase-advance of locomotor activity rhythms and peripheral clock gene expression rhythms under normal light/dark conditions [42, 43]. Another recent study showed that a high-fat diet causes a large-scale reorganization of oscillation in transcripts and metabolites in the liver [38••]. These effects are caused by both impaired CLOCK:BMAL chromatin recruitment and the induction of *de novo* oscillation of PPARγ-mediated transcriptional controls. Such reprograming of the circadian clock systems by a high-fat diet is notably reversible within a few weeks.

Several other food components also affect circadian clocks. Caffeine contained in food and drinks prolongs circadian locomotor rhythms in *Drosophila* and mice [44, 45]. The dose of caffeine required to affect circadian rhythms is ~0.05 %, which is equivalent to the dose contained in coffee. Indeed, the consumption of regular, but not decaffeinated, coffee prolongs circadian activity rhythm in mice under constant darkness [45]. Interestingly, caffeine prolongs the circadian period even in cultured cells and in mouse tissues and its analogue, theophylline, lengthens circadian rhythms in *Neurospora*, *Chlamydomonas*, *Drosophila*, and even in higher plants [44, 46–48]. Caffeine/theophylline thus might affect a fundamental part of the circadian system. A high-salt diet also affects circadian gene expression in mice. Consumption of a diet containing 4 % NaCl for more than 2 weeks induces phase-advances of clock and clock-controlled genes in peripheral tissues, whereas locomotor activity rhythms, including feeding and drinking behaviors are not affected [49]. This phase-advance effect might be similar to the powerful entrainment of liver clocks by rapidly digested starch [30], because a high-salt diet increases the expression of glucose transporters in digestive tissues and results in acute blood glucose uptake after feeding [49]. A few investigators have reported that some food components affect clock gene expression *in vitro*. Resveratrol, a polyphenol found in red wine, resets clocks and harmine, which is a harmala alkaloid found in many plants extends the period of clocks in fibroblasts [50, 51]. These findings indicate that both central and peripheral circadian clocks can be affected by consumed food factors.

## Energy State and Clocks

Several studies have shown that energy status affects circadian clock systems. The intracellular NAD(H)^+^/NADP(H) ratio that depends on the metabolic status of cells affects the ability of CLOCK/NPAS2:BMAL1 transcription factors to bind DNA [52]. Intracellular NAD^+^ concentrations are controlled downstream of clock genes in a circadian manner through Nampt, which is a gene encoding restriction enzymes for the NAD^+^ synthetic pathway that shows circadian oscillation [53, 54]. The NAD^+^-dependent deacetylase Sirt1 modulates CLOCK-mediated chromatin remodeling and PER2 protein degradation in a circadian fashion [55–57]. Another NAD^+^-dependent enzyme, PARP1, regulates BMAL/CLOCK interaction through the ADP-ribosylation of CLOCK protein [58]. These NAD^+^-dependent molecules control clocks in the feedback system. Thus, disrupting NAD^+^ oscillation alters behavioral and metabolic circadian rhythms in mice [59]. Another factor that connects cellular energy status with intracellular clocks is AMPK, which senses AMP/ATP ratios and thus acts as an intracellular energy sensor. The clock genes CK1ε and CRY1 are phosphorylated by AMPK, and this process results in the degradation of PER2 and CRY1, respectively [60, 61]. Other clock genes, Rev-erbα/β and Rorα/β are also critically involved in energy homeostasis. Rev-erbα/β is activated by heme, which reflects the cellular redox state [62], and represses Bmal1 as well as hundreds of genes related to lipid homeostasis [6, 63]. Rorα might function as a lipid sensor, because it is activated by cholesterols and regulates expression of several genes that are involved in lipid metabolism [64]. Other nuclear receptors, such as PPARs, are also important for both lipid sensing and clock function as they are activated by free fatty acids and interact with Per2, Sirt1, and PGC1α [9, 20, 65]. The activation of PPARα causes circadian behavioral changes [42], and mice lacking PGC1α have altered circadian activity and lipid metabolism [66]. Moreover, a recent study revealed the circadian cooperation of PPARs because PPARγ-dependent lipogenesis that is activated by nocturnal feeding in mice promotes fatty acid use via a circulating lipid that activates PPARα in muscle [67••]. Interestingly, a high-fat diet diminishes the rhythmic circulation of the lipid. The activity of PPARs and lipid mediators might play important roles in both circadian behavioral control and in lipid homeostasis. These findings showed that circadian clocks control energy metabolism and that energy status affects clocks as feedback loops. Nutritional changes must affect the status of circadian clocks as well as energy status.

## Timing of Food Intake Affects Energy Metabolism

The mutual influences of circadian clocks and energy metabolism suggests that feeding time critically impacts metabolism. High-fat diets induce obesity and increase risk for metabolic diseases. However, time-restricted feeding with a high-fat diet without caloric reduction suppresses obesity and metabolic diseases [68••, 69]. This remarkable effect of time-restricted feeding is probably due to fine tuning of the circadian clocks. Intake of a high-fat diet *ad libitum* attenuates the amplitude of clocks, whereas time-restricted feeding restores the amplitude. Much evidence supports the notion that feeding time affects obesity and metabolic status. Mice gained more weight when fed with a normal or a high-fat diet during 12-h in the daytime than during 12-h in the night-time [70, 71]. Mice housed under a light/dim-light cycle increased food intake ratios during the light phase gained weight and became more glucose intolerant than mice under normal light/dark cycles, despite equivalent total caloric intake and total daily activities [72]. Similarly, adipocyte-specific deletion of the Bmal1 gene increased the food intake ratio of the light phase and body weight [73•]. Ablation of the adipocyte clock altered the circulating concentration of polyunsaturated fatty acids in the hypothalamus, resulting in a change of feeding behavior. Moreover, rats fed with a butter-based, high-fat diet had an increased ratio of eating during the light phase, overate, and developed obesity [74]. These results indicate that diurnal feeding causes weight-gain in nocturnal rodents. Not only diurnal/nocturnal feeding but also breakfast/dinner timing affects body weight and energy metabolism. Weight gain, hyperinsulinemia, and hyperleptinemia were suppressed in mice fed with a bigger breakfast and a smaller dinner compared with mice fed *ad libitum* [75]. Early nocturnal fasting increased lipogenesis and resulted in an increase of body weight in mice [76], and late nocturnal fasting reduced body weight gain in rats [77]. The timing of food intake also affects body weight and the risk of obesity in humans [78, 79]. A study of two isocaloric weight loss groups found greater improvement of many metabolic markers, body weight, fasting glucose, insulin, TG, OGTT, ghrelin, mean hunger scores, and satiety scores in the group given a bigger breakfast and a smaller dinner than vice versa [80•]. Another study showed that early mealtimes significantly decreased TG and LDL cholesterol levels in serum [81]. Moreover, several human epidemiological studies have suggested a correlation between eating pattern and obesity. The frequency of breakfast was inversely associated with weight gain in a cohort of 2,216 adolescents [82]. Similarly, skipping breakfast increases the odds ratios for adult obesity, overweight children, and visceral adiposity in overweight Latino youth [83–85]. In addition to frequency, the glycemic index of breakfast might affect control of appetite and blood sugar levels in both adults and children [86]. Moreover, night eating syndrome characterized by a time-delayed eating pattern is positively associated with BMI [87]. These findings indicate that late meals and skipping breakfast leads to body weight gain and obesity in humans as well as in experimental animals.

## How Does Meal Timing Affect Metabolism?

Although the mechanisms underlying the effects of differential meal timing upon obesity remains obscure, some evidence is quite suggestive. One aspect is that unusual food timing causes overintake partly due to an insufficient satiety function. As circadian clocks control the expression of leptin that suppresses appetite [88], circadian misalignment causes a reduction of serum leptin throughout the day [89]. Indeed, a few studies have found that impaired feeding rhythms result in increased total food intake [71, 74, 76]. It is noted that leptin levels are reduced and energy intake is increased during short sleep durations in humans [90], and some epidemiological studies have associated sleep deprivation with energy intake and obesity [91]. Time-restricted feeding during the nonactive phase might cause sleep deprivation and overeating. However, some studies have shown that feeding time affects body weight even under isocaloric conditions [68••, 76, 80•]. Altered calorie expenditure might be another explanation for changes in weight. Core body temperature is controlled by circadian clocks [92], and a recent study has shown a mechanism of circadian thermogenesis. Rev-erbα suppresses and generates the rhythmic expression of Ucp1, which is an important factor in nonshivering thermogenesis in brown adipose tissues, and rhythms of body and BAT temperatures are attenuated in the Rev-erbα-knockout mice [93]. The circadian control of body temperature suggests that thermogenesis varies during the day. Indeed, diet-induced thermogenesis (DIT) elicits circadian variations in humans; DIT is highest in the morning, followed by the afternoon and night [94]. Such circadian thermogenesis could reasonably explain increases in the body mass of persons who skip breakfast. Light conditions also affect both thermogenesis and metabolism. Constant light impairs thermogenesis against cold stimuli in squirrel monkeys [95]. Four days of constant light increase food intake, decreased energy expenditure, and resulted in an immediate weight gain in mice [96]. As constant light attenuates rhythms of the central clock, disorganized feeding behavior, sleep/wake cycles, and energy homeostasis, including thermogenesis might result. In addition, a shift of light conditions or feeding time affects metabolism, body temperature, and body weight within a few days [24, 89]. These immediate effects are probably due to desynchronization among internal clocks similar to jet lag. Chronic jet lag, including shift-work, inevitably increases risk for metabolic disorders [97–99]. Intriguingly, intake of food in normal active-phase restores metabolic abnormality in rodent models of shift-work [97, 100]. Disturbed systemic cooperation among clocks might be the most critical factors in the impaired energy metabolism induced by unusual feeding. One study of early nocturnal fasting in mice found that the amplitude of lipogenic genes, such as Srebp-1c and Pparα was increased, resulting in increased *de novo* lipogenesis in the liver [76]. As mentioned above, circadian lipogenesis in the liver is drastically reprogrammed by nutritional change [38••], and cooperatively controls fat use in the muscle via the circulating lipid [67••]. Unpredictable feeding times might disturb the circadian harmonization of metabolic processes beyond organs and finally disrupt energy homeostasis (Fig. 1).

## Conclusions

Circadian clocks in animals are tightly connected to energy homeostasis and are affected by feeding time as well as food composition/components. Clocks control energy metabolism and metabolic states influence clocks. The prevalence of metabolic diseases has increased in many countries where circadian behaviors, including meal times, can be disrupted and individuals can be deprived of sleep. Time-restricted feeding or a balanced breakfast can powerfully entrain and thus amplify circadian clocks in peripheral tissues, whereas feeding at unusual times or with a high-fat diet attenuates these clocks. Light/dark cues are also important to maintain cooperation between circadian systems and energy homeostasis through the central clock. Consideration of appropriate meal times is one wise way to control metabolic diseases even without caloric reduction. The consumption of beneficial food components, such as polyphenols, unsaturated fatty acids, and fiber, at suitable times would help to promote health in the same way as medication is administered at specific times in chronopharmacology. Not only the quality and quantity but also timing is important for nutrition.
